# Genotypic discrepancies arising from imputation

**DOI:** 10.1186/1753-6561-8-S1-S17

**Published:** 2014-06-17

**Authors:** Anthony L Hinrichs, Robert C Culverhouse, Brian K Suarez

**Affiliations:** 1 Department of Psychiatry, Washington University School of Medicine, St. Louis, MO 63110, USA; 2 Department of Medicine and Division of Biostatistics, Washington University School of Medicine, St. Louis, MO 63110, USA; 3 Department of Genetics, Washington University School of Medicine, St. Louis, MO 63110, USA

## Abstract

The ideal genetic analysis of family data would include whole genome sequence on all family members. A strategy of combining sequence data from a subset of key individuals with inexpensive, genome-wide association study (GWAS) chip genotypes on all individuals to infer sequence level genotypes throughout the families has been suggested as a highly accurate alternative. This strategy was followed by the Genetic Analysis Workshop 18 data providers. We examined the quality of the imputation to identify potential consequences of this strategy by comparing discrepancies between GWAS genotype calls and imputed calls for the same variants. Overall, the inference and imputation process worked very well. However, we find that discrepancies occurred at an increased rate when imputation was used to infer missing data in sequenced individuals. Although this may be an artifact of this particular instantiation of these analytic methods, there may be general genetic or algorithmic reasons to avoid trying to fill in missing sequence data. This is especially true given the risk of false positives and reduction in power for family-based transmission tests when founders are incorrectly imputed as heterozygotes. Finally, we note a higher rate of discrepancies when unsequenced individuals are inferred using sequenced individuals from other pedigrees drawn from the same admixed population.

## Background

The ideal genetic analysis of family data would include whole genome sequence data on all family members. To save cost, a procedure has been suggested to avoid having to sequence every individual [1]. In particular, this procedure uses dense sequence data on a subset of individuals and sparse, inexpensive, genome-wide association study (GWAS) chip genotypes on all individuals to infer sequence-level genotypes on the related, unsequenced individuals. The Genetic Analysis Workshop 18 (GAW18) data providers have followed these procedures as documented in [2]. We examine the quality of the imputation to identify potential consequences for this approach.

## Methods

### The data

The GAW18 data providers obtained family data from two studies: the San Antonio Family Heart Study and the San Antonio Family Diabetes/Gallbladder Study [3]. The GAW18 data set contains GWAS chip genotypes on 959 individuals from 20 pedigrees. Of this sample, a subset of 464 individuals also has whole genome sequence data. Although four families have no sequenced individuals, the remaining pedigrees are sequenced for roughly half of their members (Table 1).

**Table 1:** Discrepancies by family

	Sequenced individuals			Nonsequenced individuals			All individuals		
**Fam_ID**	***N***	***D***	***D/N***	***N***	***D***	***D/N***	*N*	*D*	*D/N*

T2DG23				32	14678	**458.7**	32	14678	**458.7**
T2DG15				41	17431	**425.1**	41	17431	**425.1**
T2DG14				40	15459	**386.5**	40	15459	**386.5**
T2DG25				33	12714	**385.3**	33	12714	**385.3**
T2DG17	20	5287	**264.4**	22	5639	256.3	42	10926	260.1
T2DG20	20	4977	**248.9**	16	2943	183.9	36	7920	220.0
T2DG08	25	5461	**218.4**	43	9112	211.9	68	14573	214.3
T2DG27	17	3686	**216.8**	18	3074	170.8	35	6760	193.1
T2DG02	43	9108	**211.8**	43	7922	184.2	86	17030	198.0
T2DG21	19	3915	**206.1**	16	2630	164.4	35	6545	187.0
T2DG04	38	7245	190.7	25	4155	166.2	63	11400	181.0
T2DG06	39	6976	**178.9**	25	3174	127.0	64	10150	158.6
T2DG03	38	4675	123.0	39	6943	**178.0**	77	11618	150.9
T2DG11	29	5132	**177.0**	6	774	129.0	35	5906	168.7
T2DG10	40	5127	128.2	24	4058	**169.1**	64	9185	143.5
T2DG16	26	3211	123.5	22	3434	**156.1**	48	6645	138.4
T2DG47	12	1785	148.8	10	1547	**154.7**	22	3332	151.5
T2DG09	27	3182	117.9	6	878	**146.3**	33	4060	123.0
T2DG07	30	3378	112.6	6	867	**144.5**	36	4245	117.9
T2DG05	40	4349	108.7	28	3058	**109.2**	68	7407	108.9

Discrepancies in the full comparison single-nucleotide polymorphisms set between GWAS data and GENO data sets, by family, individuals sequenced and individuals imputed. Bold indicates highest discrepancy rate by subsample.

*N=*number of individuals in the family

*D=*number of discrepancies observed within the family

*D/N=*average number of discrepancies observed per family

### Generation of the data by the GAW18 providers

We will distinguish between two ways that missing data were "filled in" in the GAW18 data: filling in missing sequence data in the sequenced individuals will be referred to as "imputation," and inferring sequence-level data for individuals who were only genotyped using a GWAS chip will be referred to as "inference." We understand the imputation and inference process followed by the GAW18 data providers to consist of the following steps: (a) the GWAS chip data were phased (and any untyped GWAS chip alleles imputed) using MaCH [4], and a haplotype scaffolding for the families was created; (b) missing sequence data in the sequenced individuals were imputed using MaCH; (c) sequence haplotypes for the unsequenced individuals were inferred from the sequenced individuals using haplotype scaffolding derived from the GWAS chip data; (d) SimWalk2 was used to identify Mendelian errors and blank inconsistent genotypes; and (e) Merlin [5] was used to reimpute missing genotypes. The GAW18 data providers supplied a dosage file for each sequence variant. They did not provide other quality scores for the imputation and inference.

### Selection of single-nucleotide polymorphisms and individuals for discrepancy evaluation

The sequence data were provided in "VCF" (Variant Call Format) files. Starting with the 8,348,685 single-nucleotide polymorphisms (SNPs) that made it through quality control to end up in the final genotyping files [2], we used the VCF files to find uniquely occurring rs numbers from dbSNP that mapped to an existing SNP in the GWAS sample. The SNPs were required to map to the same chromosome and have alleles that could be "flipped" to align the strands; that is, a SNP of the type A/C in GWAS and T/G in sequencing could be aligned, but a SNP of the type A/C could not be aligned with an A/G SNP. Furthermore, because the A/T and C/G polymorphisms could have ambiguous alignment, these were discarded before comparisons were performed. Overall, this resulted in 451,279 SNPs for comparison. We will call these SNPs the "full comparison SNPs."

We compared two data sets containing these 451,279 SNPs: first, the "GENO" data set (the final, cleaned, sequence) and second, the "GWAS" data set (as provided, assembled from three different GWAS chips [2]). Because of varying call rates, we did not count missing genotypes as discrepancies between the two files (i.e., a discrepancy was noted only when a genotype was present [i.e., called] in both data sets but was not identical).

We identified one strong outlier: sequenced individual T2DG0400247 contained 11,576 discrepancies across the 451,279 SNPs. The next most discrepant individual had "only" 1880 discrepancies. The sample for individual T2DG0400247 may have somehow changed between the GWAS and the sequencing, perhaps because of a sample swap or contamination. This individual was removed from all of our subsequent analyses, leaving 958 individuals with GWAS data and 463 individuals with whole genome sequencing.

Genotyping for the GWAS was performed on several different Illumina platforms, resulting in a range of missing rates (because of different SNPs on the platforms). The SNPs called from sequencing also had a high variability in missing rate. Therefore, to have a frame of reference that avoids focusing on the GWAS genotyping process or sequencing process per se, we identified a subset of SNPs with a 98% or better call rate for both GWAS and sequencing. There were 235,549 SNPs in the "high call rate" set.

### Evaluation of discrepancies

We then examined discrepancies in four ways to help identify causes of discrepancy: by individual, by presence or absence of sequence data, by family, and by allele dosage estimate. We also divided the discrepancies by analytical process: imputation (filling in missing genotypes in sequenced individuals) and inference (inferring sequence level data for individuals without sequencing). In the sequenced individuals, imputed genotypes were determined by comparing the VCF (sequencing reads) file with the final genotype calls file, GENO. Genotypes that were missing in the VCF file for a sequenced individual but present in the GENO file were, by definition, imputed.

## Results

We first present the results for the high call rate SNPs alone and then compare these with the results found in the full comparison SNPs set. In both cases, we look at the rate of discrepancies between the GWAS file (based on a genotype chip) and the GENO file (genotype calls based on sequence, imputation, or inference).

### Discrepancy rate for the high call rate single-nucleotide polymorphisms

#### Overall discrepancy rate

The overall discrepancy rate combined across sequenced, imputed and inferred genotype calls for this SNP set was low (Table 2). For this broadest group of subjects (958 individuals) in the high call rate SNPs (235,549 SNPs), calls were present in both the GWAS and the GENO data sets 99.46% of the time. Of these, 197,984 were discrepant (0.09%) for an overall concordance rate of 99.91%. There were no discrepancies at all for 68.6% (*N *= 161,573) of these SNPs.

**Table 2:** Discrepancies by process type

Discrepancy		High call rate SNPs (98% call rate) 235,549 SNPs		Full comparison SNPs 451,279 SNPs	
Type	Subjects	Genotypes (*N*)	% Discrepant	Genotypes (*N*)	% Discrepant
Imputation	463	205,962	25.16	1,864,804	28.82
Sequencing	463	107,780,325	0.03	197,178,315	0.06
Inference	495	116,463,033	0.10	222,926,764	0.20
Inference Families with sequence	349	82,103,861	0.07	157,186,202	0.18
Inference Families without sequence	146	34,359,172	0.18	65,740,562	0.26

Discrepancies between genome-wide association study (GWAS) data and GENO data sets, divided by analytical process. "Imputation" fills in missing genotypes in sequence data. "Inference" infers phased sequence data on unsequenced individuals based on GWAS data. *SNP*, single-nucleotide polymorphism.

#### Discrepancies in the sequenced individuals

However, looking at discrepancy rate by call process yields a very different picture. As indicated in Table 2, when we compare the sequence calls with the GWAS chip calls in the 463 sequenced individual, we see a low discrepancy rate (0.03%). This accounted for the vast majority of the genotype calls for these individuals (99.8%). Imputation was used to fill in many of the missing calls in the sequenced individuals. The 0.2% of the genotype calls for these high call rate SNPs generated by imputation yielded a surprisingly high discrepancy rate of 25.2%. An analysis of these discrepancies yields some interesting results. This high discrepancy rate occurred even though the number of imputed individuals for any given SNP was very low (at most 9 of the 463 individuals). Also, the majority of these discrepancies (98.6%) consisted of a homozygote call from the GWAS chip and a heterozygote call from the imputation. We believe that all imputed genotype calls included in the GENO file were nonambiguous. This is based on the data description (which states that likely imputation errors were left blank) combined with our observation that all genotypes with nonintegral dosage values (e.g., dosage = 0.001 or 0.999), as well as many additional genotypes with integral dosage values, were blank in the GENO file.

#### Discrepancies in the nonsequenced individuals

Not surprisingly, we see higher discrepancy rates in individuals whose sequences were inferred than in the sequenced individuals themselves. Having no sequenced family members clearly degraded the process further: the discrepancy rate for inferred individuals without genotyped family members was approximately twice the rate of discrepancies found in individuals with sequenced family members (Table 2, final two rows).

### Discrepancy rate for the full comparison single-nucleotide polymorphisms

As would be expected, when we expand beyond the high call rate SNPs, the discrepancy rate increases.

#### Overall discrepancy rate

The overall discrepancy rate combined across sequenced, imputed, and inferred genotype calls across the full comparison SNPs was also low (see Tables 1 and 2). In this group (958 individuals with 451,279 SNPs, for roughly 432 million potential calls), calls were present in both the GWAS and the GENO data sets 97.6% of the time. Of these, slightly more than 1 million (1,099,402) were discrepant (0.26%), for an overall concordance rate of 99.74%. We note that a small number of these SNPs (fewer than 60) were outliers with an unusually high number of discrepancies. (Two SNPs had more than 900 discrepancies, and 8 SNPs had more than 800 discrepancies. These are likely to have been cases in which the sequenced SNP was not the SNP genotyped on the GWAS. None of these SNPs were in the high call rate SNPs. In the full sample, excluding these SNPs results in a slight decrease in discrepancies in the second decimal place [results not shown].)

#### Discrepancies in the sequenced individuals

As was seen for the high call rate SNPs, an examination of discrepancy by source of the genotype call yields a much different picture. As indicated in Table 2, for the 463 sequenced individuals, approximately 99.1% of the genotype calls in the GENO file were sequence reads. When we compare these sequence calls with the GWAS chip calls, we see a low discrepancy rate overall (0.06%). Although this is twice the rate found for the high call rate SNPs, it still strongly supports the supposition that both the sequencing calls and the genotype chip calls are highly reliable. However, attempting to impute the last 0.9% yielded a high discrepancy rate of 28.8%, slightly higher than the 25.2% rate found for the high call rate SNPs. An analysis of these discrepancies yields some interesting results. As was true for the smaller SNP set, the majority of these discrepancies (98.6%) consisted of a homozygote call from the GWAS chip and a heterozygote call from the imputation. In all of these cases as well, the evidence suggests that there was no ambiguity in any of these calls.

#### Discrepancies in the nonsequenced individuals

Not surprisingly, we see higher discrepancy rates in individuals whose sequence was inferred than in the sequenced individuals themselves. Having no sequenced family members clearly degraded the process further: the discrepancy rate for inferred individuals without genotyped family members was approximately twice the rate of discrepancies found in individuals with sequenced family members (Table 2, final two rows). A breakdown of the discrepancy rate by family and sequenced versus inferred family members is provided in Table 1.

## Discussion and conclusions

Our ability to critique the overall imputation and inference process was limited by the absence of imputation quality measures in the distributed data. For the reasons noted, we believe that only unambiguous imputation calls were included in the data. Almost all of the discrepancies between imputed and GWAS chip genotypes in sequenced individuals involved heterozygous imputation calls. Even though future researchers may be unlikely to follow this exact method, we believe our results highlight several generally applicable points: First, this is a cautionary paper. The highly skilled providers of the GAW18 data, in collaboration with one of the founders of the field of genetic imputation, provided data to the GAW participants that were unreliable in some places. Clearly, evaluating imputation quality is critical when using imputed data. However, the standard quality scores provided by the imputation programs, such as the allelic R^2 ^[6] do not take chance agreement into account, which is particularly problematic for rare variants. Nonetheless, the IQS [7], which does takes chance agreement into account, is still not as widely used as might be warranted. Although a straightforward application of the IQS requires true genotypes for comparison, multiple approximations have been suggested (e.g., [8]).

Because of the complex imputation and inference method used by the GAW18 data providers, it is unclear at what point the discrepant heterozygotes were introduced into the process. It may be that these were introduced by Merlin when calculating the probabilities of each possible genotype for missing data in the complete pedigrees. If this is the case, then a heterozygote may be computationally very likely: a pair of heterozygous founders is completely compatible with all possible offspring genotypes; deviations from Hardy-Weinberg (caused by the Wahlund effect, for example) may cause expected heterozygosity to be higher than observed; and in the case of deletions, the apparent genotyping errors that can occur from the transmission of the "null" allele can be resolved by assuming that the parent is, in fact, a heterozygote. It may be better to allow missing sequence data to remain missing and accept the inference of missing genotypes on related individuals than to use highly likely genotypes inferred from the pedigree data. This may be especially important when studying rare variants because a modest number of inferred heterozygous founders could greatly influence transmission tests.

Despite these cautions, we see clearly that the process of sequencing some individuals and inferring genotypes for related individuals produces high-quality data; this provides a very good first step in filling the gap until complete data are available. The inference process is less robust when using sequence data from unrelated individuals.

Overall, the inference and imputation process worked very well. It is clear that GWAS chip data can be phased with high accuracy and sequence data can be inferred with high concordance to the GWAS chip genotypes. There are three conclusions from our investigation: (a) there is a very high concordance rate between genotypes obtained from sequencing and those from a GWAS array. (b) When sequencing results in missing genotypes, it may be best to retain the missingness. If sequencing failed in a region because of abnormalities such as deletions, the imputation process may not have appropriate reference data to work with. This is especially true in the case of rare variants, with which incorrectly imputing a heterozygote may reduce the power or create false positives for transmission disequilibrium tests. (c) The imputation and inference process may result in final data that is discrepant from the original GWAS data. As a consequence, it may be prudent to incorporate the GWAS chip genotype calls into the final data set used for analysis (e.g., blanking discrepant genotypes).

## Competing interests

The authors declare that they have no competing interests.

## Authors' contributions

BKS designed the overall study. ALH and RCC conducted statistical analyses. All authors assisted in drafting the manuscript and approved the final version.
